# Translating global evidence into local implementation through technical assistance: a realist evaluation of the Bloomberg philanthropies initiative for global Road safety

**DOI:** 10.1186/s12992-024-01041-z

**Published:** 2024-05-10

**Authors:** Rachel Neill, Angélica López Hernández, Adam D. Koon, Abdulgafoor M. Bachani

**Affiliations:** Johns Hopkins International Injury Research Unit, Health Systems Program, Department of International Health, Johns Hopkins Bloomberg School of Public Health, 615 N. Wolfe Street Suite E8527, Baltimore, MD 21205 USA

**Keywords:** Road safety, Low- and middle-income countries, Technical assistance, International development, Realist evaluation

## Abstract

**Background:**

Traffic-related crashes are a leading cause of premature death and disability. The safe systems approach is an evidence-informed set of innovations to reduce traffic-related injuries and deaths. First developed in Sweden, global health actors are adapting the model to improve road safety in low- and middle-income countries via technical assistance (TA) programs; however, there is little evidence on road safety TA across contexts. This study investigated how, why, and under what conditions technical assistance influenced evidence-informed road safety in Accra (Ghana), Bogotá (Colombia), and Mumbai (India), using a case study of the Bloomberg Philanthropies Initiative for Global Road Safety (BIGRS).

**Methods:**

We conducted a realist evaluation with a multiple case study design to construct a program theory. Key informant interviews were conducted with 68 government officials, program staff, and other stakeholders. Documents were utilized to trace the evolution of the program. We used a retroductive analysis approach, drawing on the diffusion of innovation theory and guided by the context-mechanism-outcome approach to realist evaluation.

**Results:**

TA can improve road safety capabilities and increase the uptake of evidence-informed interventions. Hands-on capacity building tailored to specific implementation needs improved implementers’ understanding of new approaches. BIGRS generated novel, city-specific analytics that shifted the focus toward vulnerable road users. BIGRS and city officials launched pilots that brought evidence-informed approaches. This built confidence by demonstrating successful implementation and allowing government officials to gauge public perception. But pilots had to scale within existing city and national contexts. City champions, governance structures, existing political prioritization, and socio-cultural norms influenced scale-up.

**Conclusion:**

The program theory emphasizes the interaction of trust, credibility, champions and their authority, governance structures, political prioritization, and the implement-ability of international evidence in creating the conditions for road safety change. BIGRS continues to be a vehicle for improving road safety at scale and developing coalitions that assist governments in fulfilling their role as stewards of population well-being. Our findings improve understanding of the complex role of TA in translating evidence-informed interventions to country-level implementation and emphasize the importance of context-sensitive TA to increase impact.

**Supplementary Information:**

The online version contains supplementary material available at 10.1186/s12992-024-01041-z.

## Background

Road traffic crashes are the leading cause of death for persons aged 5–29 years age [1], and the 12th leading cause of deaths overall [2]. Road traffic mortality is three times higher in low-income countries than high-income countries (HICs), despite low-income countries having less than 1% of global motor vehicles [2]. Over half of traffic-related deaths are vulnerable road users (e.g., pedestrians, cyclists, and motorcyclists) [2].

Attention to road safety has grown, supported by evidence on the severity of the problem and solutions [3]. Successive ‘Decades of Action for Road Safety’ have raised awareness, and new institutions have improved policy cohesion and civil society mobilization [3]. The global road safety community has also cohered around a consensus-based solution – the safe system approach – developed in Sweden and increasingly applied globally. The safe system approach is a human-centered, proactive approach that shifts the focus of road safety from preventing crashes and improving road user behavior to preventing deaths and injuries while accounting for human error [4]. Despite global momentum, there is limited implementation of the safe system approach in low- and middle-income countries (LMICs) [3, 5, 6]. Global road safety programs emphasize the adaptation of the safe systems model to LMICs [5, 7], even though the implementation context in LMICs varies significantly [8].

### The role of technical assistance

Technical assistance (TA) is one way to increase the uptake of the safe system approach and other evidence-informed interventions. TA is a capacity-building process to design and/or improve the quality, effectiveness, and efficiency of programs and policies, [9]. Multi-country TA programs seek to translate the safe system approach to LMICs to reduce traffic-related injuries and mortality. The Bloomberg Philanthropies Initiative for Global Road Safety (BIGRS) is one of the largest and longest-standing multi-country road safety TA programs. This analysis concerns BIGRS Phase II, which provided a common package of TA interventions to ten LMIC city governments from 2014 to 2019. By the end of Phase II, cities differed considerably on the scale and scope of implementation.

BIGRS’ differential experiences across LMIC cities present an empirical case study on the feasibility of adapting common technical approaches across divergent contexts and the TA’s role. How much influence does TA have? What is the role of context in shaping TA providers’ and recipients’ agency?

A diverse body of scholarship concerns these questions and can guide empirical inquiry. Diffusion of innovation theory describes the process of transferring an evidence-informed intervention from one setting to another [10, 11] and has been used to explore TA effectiveness [9]. Diffusion of innovation theory focuses on intervention characteristics, intervention adaptation, and how adaptation influences adoption and fidelity [10–12]. Greenhalgh’s Determinants of Diffusion, Dissemination, and Implementation of Innovations in Health Service Delivery and Organization Conceptual Model builds on diffusion of innovation theory by mapping considerations that influence the uptake of innovations. These include credibility, personal relationships, effective communication,translation of the innovation to meet end-users needs, and support to adopters [12]. More broadly, social science theories consider the role of structural context (e.g., laws, social norms, and governance) and pragmatic implementation contexts (e.g., individuals, relationships, and organizational cultures) in determining adaptation, and implementation [13]. These literature bring different perspectives to explain change through the interaction of interventions, actors, and context.

However, there is limited application of this literature to understand TA, especially road safety TA. A growing body of case studies describes what works and does not work for improving road safety in LMICs [14–16]. Limited research emphasizes political will, intervention tailoring, human and financial resources for dissemination [17], the best practice exchange [18], technology transfer [19], and the power of multi-sectoral coalitions [20] to translate road safety evidence into practice. However, despite the existence of several multi-million-dollar road safety TA and funding programs [21–25], we did not identify any evidence on the role of TA in supporting or inhibiting road safety improvements – a key evidence gap.

### Study objective

This study aims to improve understanding of if, how, why, and under what conditions TA programs strengthen evidence-informed road safety programs in LMICs. We do this via a realist evaluation with a multiple case study design of BIGRS’ implementation, comparing how common TA interventions interacted with contextual factors to produce differential observable outcomes in Accra, Bogotá, and Mumbai. These findings are distilled into a program theory that provides insight into how ‘global’ approaches are translated to country-level implementation and can be used to guide TA’s design and implementation.

## Methods

Realist evaluation connects theories of ‘how the world works’ with ‘how a program works’ to explain how interventions trigger mechanisms in different contexts [26]. We used a realist evaluation methodology [27] to identify how, why, and under what conditions TA can strengthen evidence-informed road safety, with a multiple case study design to improve understanding of how BIGRS worked in diverse contexts [28, 29] [26]. This methodology was selected to identify the underlying mechanisms driving the program’s differential outcomes in different contexts [27].

### Realist evaluation

Programs are theories about how something works. They are embedded into open systems and adaptively interact with the context. Intervention outcomes result from engagement between program actors and contexts [27]. An intervention-context-mechanism-outcome pattern (ICMO) represents this [27] (Table 1).

**Table 1:** Definitions of the ICMO

Term	Definition	Illustrative example relevant to BIGRS
Intervention	Activities or resources introduced by the program [30]	BIGRS' TA supports analysis of city road safety surveillance data.
Context	The broader environment that influences the mechanism. This includes policies, processes, relationships, norms, priorities, beliefs, and resources. Context is adaptive; interventions are introduced into contexts, and contexts change because of the intervention [31]	Availability of data, quality of the data, individuals in the city who work with the data, platforms for analyzing and disseminating data, credibility of the data.
Mechanism	Underlying, generative behaviors, reasoning, or reactions of agents that occur because of intervention in a specific context and which lead to the observed outcome [26]	Results from the data analysis change the mind of city officials.
Outcome	Observable pattern of behavior or implementation [26]	City officials use the data to target their own road safety interventions.

We adhered to the Realist and Meta-narrative Evidence Syntheses: Evolving Standards (RAMSES) II reporting guidelines for realist evaluation to guide design, data collection, and analysis [26], provided in Additional File 1. The study protocol is in Additional File 2.

### Study setting

Bloomberg Philanthropies’ BIGRS Phase Two was implemented in Accra, Addis Ababa, Bandung, Bangkok, Bogotá, Fortaleza, Mumbai, Ho Chi Min, Sao Paulo, and Shanghai from 2014 to 2019 and is the focus of this study. BIGRS is currently in its third phase and has scaled up to 27 cities and two states across Latin America, sub-Saharan Africa, and Asia.

Cities applied for BIGRS-supported TA by submitting a proposal that demonstrated their commitment to and plans for road safety. This is important because it meant that cities demonstrated a common commitment and desired TA, at least in theory. Funding for interventions (e.g., re-designing an intersection or mass media campaigns) came from city governments.

BIGRS’ TA came with a technical agenda – aligned to the safe system approach – on how road safety should be improved. BIGRS’ scope was tailored to city needs within pre-existing parameters and excluding funding for capital construction. To provide TA, BIGRS seconded staff into leading road safety agencies to build institutional capacity for change. Embedded staff supported BIGRS interventions, provided direct TA to city counterparts, and often, provided cross-cutting support to city officials. In addition, seven international partner organizations managed technical activities. Partners and embedded staff were aligned to technical areas and often worked with different counterparts (e.g., an enforcement partner working with the police, an infrastructure partner working with an engineering unit).

### Case design and sampling

A multiple case study design was utilized; see Additional file 3 for details. Only cities continuing into BIGRS Phase Three were eligible for selection to ensure access to informants. We purposefully selected three cities – Accra, Ghana; Bogotá, Colombia; and Mumbai, India – with different baseline characteristics described in Table 2.

**Table 2:** Overview of cases by selection criteria

Selection criteria	Accra, Ghana	Bogotá, Colombia	Mumbai, India
**Geography** [32]	sub-Saharan Africa	Latin America and the Caribbean	South Asia
**Income-level** [32]	Lower-middle income	Upper-middle income	Lower-middle income
**Population density (2014)** [33]	4300 persons/km^2^	16,600 persons/km^2^	32,300 persons/km^2^
**Vehicles per capita, national (2014)** [34]	30 per 1000	71 per 1000	18 per 1000
**Governance for road safety across level of government** (program documents)	Centralized at the national level	Relatively decentralized to city level	Relatively centralized at the state level

### Data collection

Key informant interviews (KIIs) were our primary data source, and documents were secondary. Program documents were used to build an initial program theory, develop the interview guides, and follow ‘hunches’ about how an intervention worked in a context [35, 36]. We also snowballed documents from interviews to confirm and triangulate interview findings.

### Key informant interviews

We used a theoretical sampling approach to select informants based on their ICMO potential [27]. We iteratively sampled informants until saturation – when interviews provided no new insights [37]. Table 3 describes the KII sample. Road safety governance models influenced the balance of KII types. Road safety governance in Accra and Mumbai is more diffused than Bogotá, which had fewer government informants. KIIs also varied across cities due to differential access to informants. To overcome this disadvantage, we triangulated findings with the document review.

**Table 3:** KII sample, by case and informant type

	Informant type				
Informant Geography	Government	BIGRS staff^**a**^	BIGRS partner or collaborator	Journalists	Total sample
Accra, Ghana	7	10	4	2	23
Bogotá, Colombia	2	9	6		17
Mumbai, India	4	10	5	1	20
‘Global’ or ‘regional’ staff		4	8		12
**Total Sample**	**14**	**30**	**24**		**68**

^a^ One person in Mumbai and two people in Accra were interviewed twice (once at the beginning of data collection and then again towards the end); all other participants were interviewed once

Interviews were conducted from January 2020 to November 2022 by two members of the research team with doctoral-level training in qualitative methods. Participants were contacted via email and invited to a one-hour interview on barriers and enablers to BIGRS and mechanisms associated with program outcomes. Interviews in Mumbai were conducted via Zoom. Interviews in Bogotá and Accra were conducted in-person and on Zoom.

A realist approach to interviewing was used to build an iterative understanding of how the program worked, test our interpretations, and seek alternative explanations (Additional file 3). Interviews were recorded and transcribed with permission. Fifty-one interviews were conducted in English, recorded, and transcribed. Seventeen interviews were conducted in Spanish by a native speaker, recorded, transcribed, and translated into English by a certified translator.

### Analysis

**Fig. 1:**
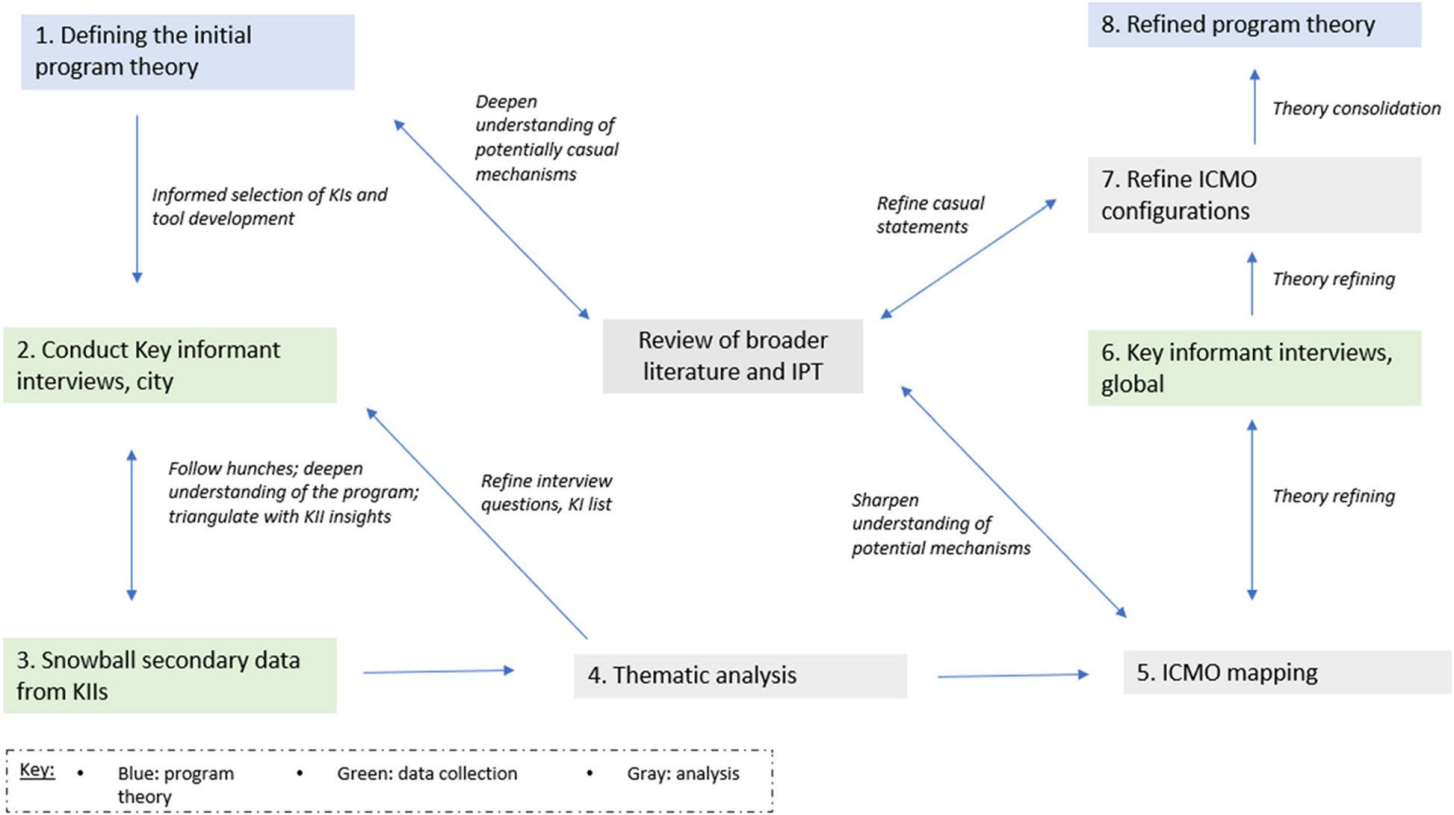
Iterative analysis process

Data collection and analysis were done iteratively using a process of retroduction [35, 36, 38] (Fig. 1).

As is common in this literature [39], BIGRS’ theory of change (TOC) was used as the initial program theory (IPT). Following guidance on realist evaluation analysis [38], we iteratively identified ICMOs and compared them to the IPT and broader literature to develop the program theory. This included an initial thematic coding of the data, a second round of theory refinement coding where themes were split into ICMOs, triangulation of findings from the documents and interviews, and comparison of the findings with existing theory to deepen our understanding of plausible mechanisms. Additional file 3 describes this process in more detail. We conducted this analysis in NVivo12.

Regular discussions were held across the research team to define and iterate on the codebook, discuss emergent themes, and review ICMO configurations. Memos were developed in Microsoft Word and documented ICMO iterations. Draft findings were shared with a subset of the participants for feedback and validation before program theory finalization.

## Results

In 2014, BIGRS initiated a common TA program in Accra, Bogotá, and Mumbai. TA interventions, individuals providing and receiving TA, the city context, and the national-level road safety context influenced implementation. Table 4 outlines interventions and outcomes. Interventions are grouped under two outcomes: (1) improved road safety capabilities (via capacity building and data) and (2) increased the uptake of evidence-informed road safety interventions (via infrastructure, enforcement, and policy support).

**Table 4:** Comparing the case studies across the intervention categories^a^

BIGRS interventions	Accra outputs and outcomes	Bogotá outputs and outcomes	Mumbai outputs and outcomes
**Outcome 1: Improved road safety capabilities**			
*Intervention 1: Capacity building on the safe systems approach*			
Trained engineers, supporting infrastructure re-design	X	X	X
Trained academic institutions to collect road safety data	X	X	X
Trained police on general enforcement policing and the police’s role in road safety	X	X	X
Trained journalists on road safety concepts and reporting	X	X	X
*Intervention 2: TA road safety data generation, analysis, and use*			
Data collection, management, and/or analysis of surveillance data	X	X	X
Annual road safety reports	X	X	X
Risk factor prevalence studies	X	X	X
Infrastructure assessments	X	X	X
Data use for decision making	X Examples: - Development of the Accra Pedestrian Action Plan - Transformation of La Paz intersection	X Examples: - Increased speed enforcement - City-wide reduction in the posted speed limit	X Example: - Incorporating pedestrian safety into infrastructure re-design projects
**Outcome 2: Increased the uptake of evidence-informed road safety interventions**			
*Intervention 1: Implemented a general deterrent roadside police enforcement model*			
Donation of equipment required for enforcement	X Examples: - Donated breathalyzers - Donated speed detection devices	X Example: - Donated nighttime speed detection devices	N/A Police had required equipment to conduct enforcement
Support to enforcement operations	X Example: - Speed and drink driving enforcement piloted via a pilot task force but did not scale beyond the pilot.	X Example: - Speed enforcement operations during pilot of speed management corridors - Police increased speed enforcement following change in speed limit.	- Barrier: - The city's priority was an automated enforcement model; no enforcement operations were implemented.
*Intervention 2: Re-designed infrastructure using a safe systems approach*			
Improving street design	X Example: - Built a pedestrian bridge at La Paz, a high-crash area *(*via *BIGRS small grant support)*; city did not fund additional large-scale infrastructure changes during Phase Two	X Examples: - Tactical urbanism interventions to reduce speed - Plazas project to improve pedestrian safety and mobility.	X Examples: - Re-designed approximately 50 intersections using safe systems approach - Launched the Mumbai street lab initiative to source infrastructure re-design plans from Mumbai urban design firms *(since ended).*
Providing technical guidance to incorporate evidence-informed interventions into existing city infrastructure projects and/or maintenance efforts	X Example: - Improved signal timing, signage, and street lighting for pedestrians and in school zones.	- Barrier: - BIGRS had limited connections to the agency responsible for existing city infrastructure and maintenance.	X Example: - Provided design inputs to some existing city projects to improve pedestrian safety and parking.
*Intervention 3: Strengthened road safety policies*			
Small p policy change (e.g. plans and guidelines)	X Examples: - Established the city’s first, multistakeholder road safety council. - Developed and implemented the Accra Pedestrian Action Plan.	X Example: - Supported the adoption of a Vision Zero agenda, making Bogotá the first LMIC city to do so.	- Barrier: - Unclear road safety governance and authority at the city level limited city-level planning efforts.
Big P policy change (e.g. regulations and legislation)^b^	- Barrier: - Road safety regulation and legislation is made at the national level.	X Example: - Changed the city-wide speed limit to the WHO-endorsed 50 kmph.	- Barrier: - Road safety regulation and legislation is made at the state and federal levels.

^a^ this table is a summary of results and is subject to change during validation

^b^ BIGRS supported national-level policy initiatives in Colombia and India during Phase II; however, this study focused on city-level policy change as this was BIGRS’ primary focus

We present one example per case that demonstrates how different interventions worked together to achieve different outcomes in case study cities. Interventions (i), mechanisms (m), contexts (c), and outcomes (o) are denoted in the text. Reference to KII data is provided as M# for Mumbai, B# for Bogotá, A# for Accra, and G# for KIs working across multiple cities. 

### Transforming junctions on Mumbai’s congested streets

When BIGRS began, Mumbai’s road safety officials used high-level figures on traffic fatalities supplemented with national or state-level statistics to guide road safety decision making *(c)*. The city-specific data required to target road safety interventions was buried in paper-based police records of variable quality *(c)*. A government official in Mumbai describes:

"*That is [a] very big [problem] because we are not like other countries, we are not getting the data correctly.”* – M17

In response, BIGRS’ TA first sought to improve surveillance data. An embedded surveillance coordinator partnered with the police to catalog and analyze city surveillance data and package it in new annual city road safety reports *(i)* [40]. Infrastructure assessments *(i)* further demonstrated how specific road junctions contributed to injuries and mortality *(o).* BIGRS staff described the change in data availability:

*“[Before] there were no reports at all […]. Now I have […] a 40-page report that talks about who the road users are, […] a list of high-risk junctions and corridors […], a map that details the hotspots where crashes are occurring […] which vehicle is causing maximum crashes, […] the time of the day, the month of the year, the day of the week.”* – M8

New data demonstrated that half of traffic-related crash victims were pedestrians, which was further reported by local city media [41, 42]. Providing granular, city-specific data shifted the focus *(m)* of government towards pedestrian safety (M8, M12, M17). The same BIGRS staff member described:

*“The government didn't know that so many pedestrians were dying in crashes. These reports help bring that to light. And when that came to light, they started taking a more serious approach.”* – M8

The emphasis on pedestrians was echoed by city government officials, who agreed that the data was illuminating (M12, M17). But the city also required solutions for this perception shift to lead to concrete action. The same government official describes the challenges *(c)*:

*“We lack the best instrument in the old system to make the road elevated, or a road underpass is very difficult because the traffic on that high main road. […] That is very critical because it we are really facing problems.”* – M17

BIGRS provided the safe system approach as a solution– but how would it work in Mumbai? This question was a central concern in all cities, especially in densely populated Mumbai, where participants described the street as a ‘contested space’ (M9, M2, M21) *(c)*. Implementing the safe system approach in Mumbai required a complex adaptation process (G5, M2, M9, G6, M7) *(i)*. A BIGRS infrastructure partner described:

*“We’re constantly trying to balance Global Best Practices versus what can be done in an Indian city while pushing boundaries to be able to think outside the box. […] it's helpful to show International Best Practices, but also at the same time, balancing it out with what’s actually possible in Indian cities.”* – M9

Implement-ability was top of mind for city government officials taking risks by trying a new approach *(c)*. BIGRS staff had to recognize those risks and work collaboratively with city government counterparts to understand how international approaches could work within local realities (B15, M13) *(c)*. A BIGRS staff member described this:

*“[When you introduce international examples], there are a lot of questions and pushback saying, ‘how could this be done [here]? That was also instructive to us. How do you deal with such situations?”* – M13

Short-term demonstration projects – for example, temporarily changing traffic flow using cones and other local, low-cost materials *(i) –* allowed city government counterparts to see the safe system approach in action on their street in a low-risk context, demonstrating that a new approach was possible (B15, M11, G7, M9, B7, M17). A city official describes:

*“People are generally not aware of the things [happening outside India], [but] the problems are same. […] That can be taken only if you can show them the models […] because firsthand information from those people is much more important.”* – M17

Seeing the possibility of change was perceived to shift the focus of road safety towards vulnerable road users and especially pedestrians (M18B, B15, M11, G7, M9, M15, M18, M17, M2, G2, M9, M7, M14, M8) *(m)*. It also created a ‘how-to’ moment, enabling city government counterparts to internalize both the concept and implementation feasibility *(m).* A city official describes what he learned:

*“[I learned] new technical things, that might be there's been a certain technical change in junction design or in the road design. […] we were not able to do that thing nicely already […] We were able to grab that opportunity properly.”* – M18

Once city government counterparts understood the potential of the safe system approach *(o)*, BIGRS TA worked with city officials to use the data and select specific junctions for re-design *(i)*. Data was critical because it helped target infrastructure improvement to junctions with an outsized number of crashes. A BIGRS staff described:

*“Now [Mumbai city government are] not just randomly doing the interventions. They're very focused on where crashes are occurring, who the victims are, who the perpetrators are, and how to ensure that these crashes don't occur at all.”* – M8

Once the mechanism for change was triggered, transforming junctions started with a pilot *(i).* Pilots ensured that the safe systems approach was feasible and appropriately adapted to the context, that its impact on different road users was understood and planned for, and that the re-design successfully reduced crashes [43–45]. Pilots also allowed the city government to understand public sentiment about the changes *(c)* [43]*.* If the public was supportive, this reduced the risk to city officials trying a new approach* (m)*. The city government counterpart’s confidence in new approaches grew *(m).* This was further reinforced by data showing that infrastructure redesign positively impacted traffic flow [43]. A pilot’s success was described as leading to exponential growth in implementation (M21, M1) [44, 46, 47]. A BIGRS partner described:

*“If you see our work, it has exponentially grown in impact. […] From that one [pilot] corridor, we […] build a relationship and trust […] and so we got a chance to do design in the intersection. Then you try it with temporary sort of barricades and then it became a big thing. And then one thing just kept leading to another to another.”* – M21

As implementation took off, BIGRS engaged local media to spread awareness about the junction transformations *(i)* (M11). After seeing firsthand what could be accomplished, the city government also committed to improving high-risk intersections in the city. However, despite growing momentum and support from both city government and city engineers to transform individual junctions, the bottom-up pilot approach presented practical scale and sustainability challenges despite this government commitment (M9, M13, documents) *(c).* A BIGRs partner described:

*“It's a challenge at times, when the city does not have the funds allocated in that year. If you do manage a successful pilot and the city takes on doing it, then it's great because they can be scaled up. But in many cases […] pilots are sort of left as just that.”* – M9

BIGRS partners described city government approvals as challenges preventing scale. In contrast, city government participants urged respect for government processes and timelines, which they saw as paramount to success *(c).* In managing these processes, city officials also took on significant work to enable each infrastructure re-design (M12, M18) – a contribution that often went unacknowledged *(c).*

### Comparison of Mumbai’s infrastructure experience with other BIGRS cities

The Mumbai infrastructure example is illustrative of common dynamics. In Bogotá, capacity building was similarly perceived as successful when it used hands-on components specifically relevant to the participants (B15, G2, B9, B7, B14), and when facilitators used a coaching model that emphasized the participant’s experience (B15, B5, B9, G2).

Accra’s and Bogotá’s infrastructure TA were also targeted at bottom-up approaches (G6, A9, A5, A6, A8, M9) and guided by city-specific data *(i*), but with limited scale. In Accra, BIGRS focused on low- or no-cost interventions (e.g, changing signal times for pedestrian crosswalks, widening pedestrian medians *(i)*) (A5, A6, A8) because the city did not control the infrastructure budget and could not budget for new interventions. BIGRS also worked with the city to re-design the infamously dangerous Lapaz intersection to improve pedestrian safety, which was funded directly by BIGRS via a small grants program *(i)* [48]. In Bogotá, tactical urbanism demonstrated speed-calming measures, and feedback from road users was gathered *(i)* (B2). However, despite promising pilots, the lack of BIGRS’ ability to influence upstream changes to road procurement tenders and design guidelines limited the scale of infrastructure outcomes in each city.

### Enforcing road safety legislation in Accra

In Accra, road safety legislation existed but needed to be enforced *(c).* In the words of a national road safety agency staff*, “there’s no real commitment in solving some of these things.”* (A17). BIGRS’ enforcement interventions started with relationship building *(i).* A BIGRS partner describes:

*“How important it is to have this relationship with the high-level police officers. Because we cannot just go to a city or road police agency and say that this is what we want to do.”* – A22

Trainings on the safe systems approach *(i)* and evidence-based enforcement operations were enabled by leadership support from the Superintendent of Police (A4) and the Mayor of Accra who championed road safety and several BIGRS initiatives (A4, A6, A3, A5, A8, A1, program documents) [48, 49] *(c)*.

However, translating training into implementation quickly stalled because the police force required equipment and certification for implementing enforcement operations *(c)* (A1, A4, A6, program documents)*.* BIGRS’ partners then donated new drink-driving and speed enforcement equipment under the condition that the city utilized the equipment to conduct enforcement pilots *(i)*. These donations were accompanied by training and certification processes (A1, A4, A25, program documents) *(c)*.

While the lack of equipment could be directly addressed by BIGRS, the disconnect between city-level enforcement efforts and Ghana’s centralized policing structure could not be so easily overcome. City police did not have the authority to conduct enforcement operations *(c)*, so in exchange for the donated equipment, the police formed a dedicated tri-partite pilot task force with the authority to use the donated equipment in a series of roadside speed and drink-driving enforcement operations *(i)*.

New training and improved accuracy of the equipment were perceived to reduce conflict between police and citizens during enforcement and improved transparency in the enforcement operations (A1, A4, A25, program documents), reducing the perception of risk of public blowback *(m).* A high-ranking police officer describes the perceived increase in acceptability from the public:

*“they don’t complain, they go to the court […] because you’ve told us that the device arefor enforcement operations (A4, A25) limiting further […] the very latest speed device, speed detection devices [equipment] because we’ve told the whole world about it.”* – A25

The collective intervention – piloting the enforcement approach, supported by training and in tandem with appropriate equipment – was also received positively by the police. A senior police officer described a shift in focus towards ensuring road safety *(m):*

*“What I’ve realised is, what a positive impact on our capability to be able to ensure road safety. [..]. With the devices, we can go to the route when they see us, all cars, cars you know approaching the robot, reduce their speed and that has really resulted in a lot of improvement.”* – A25

However, while the pilot taskforce did conduct enforcement operations, a series of upstream barriers prevented the taskforce from scaling up. Most practically, the police force still lacked dedicated vehicles for enforcement operations (A4, A25) limiting further implementation. More broadly, the social and political context (A1, A4) *(c)* remained unconducive to enforcement. A BIGRS staff describes:

*“During [the enforcement pilot], we did a special round of data collection for speed, and the data showed that there was a reduction in speed. However, the moment could not be sustained. Some of the feedback they got from the police was that [the] police could not boldly or fearlessly enforce.”* – A1

Another challenge was that the required authority to change enforcement practices was vested in national agencies instead of the city government, limiting the ability of city police to institutionalize new enforcement operations (A4, program documents) *(c).* Finally, the transfer of police was described as a challenge to sustaining enforcement interventions (A4, A2, program documents) *(c).* A BIGRS partner described:

*“We can work with person, everything agreed, and then just before we roll out, he's been transferred or there's a rotation, and we have to change everything.”*— A22

### Comparison of Accra’s enforcement experience with other BIGRS cities

Across the enforcement TA provided in the three cities, building trust with senior police officers was repeatedly emphasized (A4, B4, M16, G7, G10). Using former senior police officers from other countries was seen as key to building that neccessary trust (B4, M16, G7, G10) *(c).*

Like Bogotá, Accra’s enforcement interventions took place within broader city road safety prioritization *(c),* and BIGRS donations ensured police had the right equipment *(i)*, leading to increased enforcement (B8, G12, A4, A1, A22, A25) *(o)*. However, Bogotá’s enforcement was described as widespread and sustained (B8, G12), while in Accra, enforcement operations remained limited *(o).* The authority of the police to conduct enforcement was the key difference *(c).*

In contrast, India was moving towards an automated speed enforcement model, which contrasted with the model proposed by BIGRS *(c)*. Although automated and roadside enforcement co-exist (and they did in Bogotá), BIGRS’ roadside enforcement model did not align to the broader policy agenda in Mumbai and was not implemented.

### Reducing city-wide speed limits in Bogotá – an example of policy change

Before BIGRS, improved mobility had been the focus of several consecutive city administrations *(c)*. A BIGRS partner described the favorable baseline environment:

*“Bogotá has been concerned about road safety for a long time. [Bogotá] already had a Road Safety Directorate; […] there was already a direction with a super great team. It was easy to work in Bogotá because institutionally, they were already armed.”* – B13

During Phase II, a new Secretary of Mobility with a public health background further elevated road safety in city administration *(c)* which was perceived as critical to the city's subsequent policy change (B1, B11, B12, B13, B14, B15, B16). A city official explained:

“*It is about setting priorities. So, we [the secretariat], from the first day, said the priority is road safety, and we will do everything possible to make it so.”* – Bogotá 2

Alongside a change in government, BIGRS also hired new embedded staff, some who were former members of city government, all who were local to Bogotá, and all who were passionately committed to improving road safety *(c)* (B12). However, support for road safety did not immediately translate to speed. Instead, city officials were interested in reducing drunk driving and were explicitly resistant to tackling speed (*c)*. This was due both to concerns that reducing speed would increase traffic and also a perceived lack of concern from the population over speed (G12, G5, B8, B12). A BIGRS staff recalled:

*“Even when communicating to [the] Mayor, he had the issue of road safety in his heart the main thing he communicated and did not want to do. ‘Do not slow down on arterial roads’”* – B12

However, BIGRS’ analyses of city data *(i)* identified that speed was a serious concern on arterial roads at night (B8, B12, G5, G8). A BIGRS partner in Bogotá described:

*“The first thing I did was share with the Police the data that clearly showed that most of the deaths occurred at night or early in the morning when most roads were empty.”* – B8

This was further demonstrated by a modeling study *(i)* showing both the relationship between speed and the crash rate and that the change in speed limits would not impact average travel times. This study was important evidence, which was only possible because the city’s existing speed detection infrastructure provided the modeling data (B12) *(c).*

The presentation of this novel information to city officials was perceived to shift the focus of city officials by demonstrating that speeding was prevalent at night when roads were empty, and that reducing speed wouldn’t worsen traffic *(m)*. City officials used this data to select five arterial road corridors with high speeds, crashes, and deaths to pilot a reduced speed limit of 50 km per hour (kmph) *(o and i)*.

The speed reduction pilot required close collaboration between the Secretariat of Mobility and the police to conduct nighttime enforcement *(c).* However, the police lacked necessary nighttime radar equipment *(c)* (B8, G12), a gap subsequently filled by BIGRS’ donations *(i)*. TA was provided for the police to use the equipment and to conduct safe nighttime operations *(i),* increasing enforcement campaigns in the pilot speed management corridors *(o).* A BIGRS staff described:

*“ [It] was clear when you make enforcement operations visible, like speed enforcement down that avenue. In a matter of months, we already saw a reduction [in speed].”* – G12

The new roadside enforcement was complemented by automated speed detection cameras (*c)*; however, the public was skeptical of the speed cameras' threatening the pilot’s success *(c).* Public messaging campaigns were therefore developed using city data to demonstrate the rationale behind the speed reduction and enforcement (*i*). A BIGRS staff described:

*“Legitimacy has to do with road users' acceptance of this type of control. […] What decisions were made? Make visible the places where photodetection cameras are installed. They were published on the website of the Ministry of Mobility, and there was a strong media drive to make these cameras visible and associate the cameras with the issue of life-saving cameras.”* –B8

BIGRS also provided monitoring and evaluation support *(i) which* quickly demonstrating the pilot’s effectiveness *(o).* A BIGRS staff described:

*“In a matter of months, I already saw a reduction [in deaths]. That gave the Secretary of Mobility the confidence, trust like, ‘OK, like this is working, we are reducing deaths where we are not messing up traffic. Let's do it.’”* – G12

A city official recalled the importance of the pilots:

*“Yes, yes, yes, that was very well done. The expressive power of those corridors, of the first ones”* – B14

Because of the positive pilot results, the city increased the number of corridors with lowered speed limits *(o).* The results of the pilot were also shared with the public, reinforcing the message that the speed reduction corridors were lifesaving interventions (G12, G5) *(i)* and further reducing the perception of risk in lowering the speed limit by building public support *(m).* As the pilot gained increased support, city counterparts used the data to develop a technical document justifying the lowered speed limits to Bogotá’s city council. A BIGRS staff described:

*“To be able to argue before the City Council, it was necessary to argue with objective judgment elements […] Why did they decide to slow down? Not because it occurred to us. No, the speed was lowered because this technical document allows us to support making that decision.”* – B8

Aided by the pilot’s success and with the support built through public messaging campaigns, the city council maintained the 50 km/h speed limit on the pilot corridors *(o).* However, the city council initially did not have the authority to change city-wide speed limits permanently (c), preventing scale-up until a window of opportunity opened in 2020. During the 2020 COVID-19 pandemic, a state of emergency was declared, giving temporary executive authority to the Mayor *(c)*. Although the Secretary of Mobility (the champion of the pilot) had changed, their successor became a new champion. They successfully argued that the speed limit reduction was preventing traffic crashes, thereby reducing non-COVID-19 health emergencies and freeing up healthcare capacity during the pandemic. This allowed the Mayor to extend the speed reductions city-wide in alignment with the WHO’s advised 50 km/h *(o).*

Reflecting on Bogotá’s experience with BIGRS, a city official described how BIGRS’ comprehensive TA approach was important in supporting the city’s road safety vision:

*“We wanted to build how this systemic vision of approaching the problem. And then Bloomberg supported us with communications, technical, infrastructure, traffic calming, and enforcement issues.”* – B2

City officials and BIGRS staff alike credited city leadership for continuously supporting road safety throughout several administrations and for giving political support to technical staff who brought changes to the city *(c).* One government official commented:

“*Everyone, I think, without exception, has supported this work. I believe that the first requirement to choose a city is that there is willingness. What has been in Bogotá, really, is the political will of the leaders to carry it out. Without it, you do nothing*.” – B14

### Comparison of Bogotá’s policy experience with other BIGRS cities

The scale of change in Bogotá’s road safety programming stands apart from the other case studies. Second to this was Accra; the city government formed a new road safety council and developed the city's first Pedestrian Action Plan *(o).* Like Bogotá, BIGRS in Accra leveraged city prioritization for road safety and provided city-specific evidence *(i),* which focused city stakeholders’ efforts on the importance of pedestrian safety (A8, A1, A5, A3, program documents). Also, like Bogotá, the Mayor was a champion who lent convening power to the development of Accra’s action plan (4, A6, A3, A5, A8, A1, program documents) *(c)*. The Accra Pedestrian Action Plan was further perceived to improve coordination of different road stakeholders towards a common goal (A8, A1, A5, A3, A6, program documents).

In Mumbai, in contrast, BIGRS staff and partners described a lack of an individual champion with the authority to advance road safety policy and planning at the city level as a key challenge (M10, M11, M12, M13, M14, M15, M21).

### Revised program theory

The revised program theory for BIGRS should be considered an initial attempt to synthesize across both positive cases (where outcomes were observed) and negative cases (where outcomes were limited by specific factors) to distill a set of higher-level statements about how BIGRS works at the city level and the contexts that enable or constrain its success.

The first program theory is improved road safety capabilities, focused on capacity and data use interventions described by BIGRS staff and partners as precursors to implementation in each case study city.

**Program theory for improved road safety capabilities:**

Providing TA to increase capacity and data use *(i),* if delivered via trusted and credible TA providers who provide hands-on coaching support tailored to city needs and with counterparts interested in engaging with road safety, can strengthen road safety capabilities *(o)* because it shifts the focus of city officials towards evidence-informed approaches and creates a how-to moment to improve road safety through the safe system approach *(m).* This outcome is enabled by city prioritization of road safety *(c)* and can be disrupted if city government officials change *(c)*.

The second program theory is increasing the uptake of evidence-informed implementation of road safety interventions. In this theory, capacity building and data now comprise the necessary context that supports the interventions, and BIGRS and city officials are characterized as working together to implement.

**Program theory for increasing the uptake of evidence-informed implementation of road safety interventions:**

If trusted and credible TA providers, working with and through city champions *(c),* undertake a successful pilot *(i),* guided by city-specific data that targets interventions *(c)*, and with facilitation of city implementation via dedicated equipment, training and other supportive resources *(i)*, then this can increase the uptake of evidence-informed road safety interventions *(o).* This occurs because a pilot builds confidence that the safe systems approach is feasible in a specific road context *(m)*, and it reduces the perception of risk in adopting a new approach *(m)* by allowing city officials to gauge public sentiment. The scale and sustainability of the outcome(s) are determined by the city’s existing prioritization of road safety, the authority of the individuals and road safety agencies targeted in the intervention, and existing socio-cultural norms *(c)*. It can be disrupted if city government officials change *(c)*.

## Discussion

BIGRS’ interventions sought to accelerate cities’ adoption of the safe system approach. What united city officials were two questions – will it work here, and how? To answer those questions, TA needed to go beyond recommending that a safe system approach would work, to demonstrating how it could work, to prove that it worked (without provoking negative reactions from the public).

### How did TA work?

TA provider credibility and ability to navigate the city context were important. This was demonstrated by embedded staff who continuously connected the evidence-base and resources of international partners with the tacit knowledge and goals of city agencies. By playing a dual ‘insider-outsider’ role, embedded staff worked to create a favorable context for interventions and made interventions a better fit for the context. This describes the role of boundary-spanners who bridge insider and outsider roles to facilitate the adoption of an intervention [12].

How TA was provided was also essential. TA providers needed to understand the context and work effectively within it, not against it. Capacity-building activities needed to follow a coaching model, amplifying the existing knowledge, needs, and priorities of decision-makers. Interventions needed to be immediately relevant to the context, or TA providers risked losing credibility. BIGRS embedded staff and partners based full-time in the city again had the advantage here. This finding aligns with calls for TA to be context-sensitive [50, 51] and aligns with the characteristics of successful change agents [12].

### Why did TA work (or not work)?

The mechanism ‘shifting the focus’ was about data. Aligning with diffusion of innovation theory, data framed a ‘felt need’ for change [52] in all cities to different degrees. Bogotá was an early adopter; new data was presented within the context of political commitment to road safety, and pre-existing automated enforcement infrastructure enabled BIGRS to develop data-driven machine learning models to predict the results of the speed enforcement pilot. In Mumbai, in comparision, most of BIGRS’ Phase II activities focused on building city data capabilities to catalyze this shift in focus. ‘Shifting the focus’ was further enhanced by city officials’ ability to establish fora for governing the use of data to support policy decisions, consistent with international norms [53].

But ‘shifting the focus’ was also directly facilitated by BIGRS, making it the most uncertain mechanism. An alternative conceptualization is that BIGRS ‘shifted the focus’ by dedicating resources to specific interventions, informed by its data, which the city endorsed.

The second mechanism, creating a ‘how-to moment’, comes from diffusion of innovation’s knowledge phase [52]. Adopters must understand how an innovation works, especially if the innovation is complex [52]. Pilots allowed officials to see change in action, built confidence, and reduced the risk of stakeholder discontent from changing the road environment [12, 52]. BIGRS also had an advantage; infrastructure re-design and enforcement are trial-able approaches with quickly observable outcomes which supports innovation adoption [10, 12].

### Under what conditions did TA work (or not work)?

Moving from the first program theory outcome (‘strengthened road safety capabilities’) to the second (‘increasing evidence-informed interventions’) required more than triggering individual-level mechanisms. To change implementation, individual-level mechanisms had to translate into institutional actions by city officials– e.g., approving pilots, allocating resources, and implementing interventions. It was here that context was critical.

City champions were key to enabling change. Champions are important in diffusion of innovation theory [12] and were critical here. However, following structure-agent theory, city champions could only change areas within their control [54] and their agency varied. Comparing Bogotá and Accra is instructive. Bogotá had considerable latitude to change road safety practices, while Accra’s pilot task force failed to scale due to limited institutional and normative authority to enforce legislation. Officials in road safety agencies lamented this alongside BIGRS staff, suggesting that the interventions were compatible with the context [52] but that the city's agency was constrained.

Structural, or outer, contexts therefore determined the feasibility of converting individual and city level mechanisms into outcomes. Diffusion of innovation theory considers that innovation may not be ‘compatible’ with the context or that the system may not be ‘ready’ for change, which was important in these cases. But more important, however, was how the innovation was introduced, who introduced it, the city's priorities, and city’s authority to adopt the innovation. This points to a critical consideration – if the dissemination approach of how the innovation is introduced is incompatible with structural context, adoption will be slow or unsuccessful (even if the innovation itself fits the context).

Boundary spanning – crossing boundaries to negotiate interactions and translate knowledge from different settings [55] – is one way to bridge the gap between proposed solutions and local contexts. A 2017 multi-county nutrition project found that boundary spanning was feasible and useful to navigate context-specific challenges [56]. Our study suggests that boundary spanning – if those doing the boundary spanning are deeply embedded within the local context – could be a useful model for delivering TA. Engaging boundary spanners from the beginning to work with city government officials to design TA programs around local problems and priorities, rather than providing both with a model from elsewhere to adapt, is a practical way to design more context-sensitive TA and surface local innovations [13].

### Strengths and limitations

The goal of this study was to learn from implementation experience and develop a program theory. We did not quantitatively measure outcomes, a limitation. To improve trustworthiness, we triangulated findings across cities and data sources. However, outcomes were mainly validated with informants due to a lack of access to documents across BIGRS partners, creating some uncertainty. Another limitation was the overrepresentation of BIGRS staff and partners in our sample as compared to government officials and other city stakeholders. The reasons for this were both practical – e.g., scheduling interviews over Zoom, governance differences across cities – and representative of broader findings – government official turnover limited available informants. Finally, several authors (but not the first author) were involved in BIGRS’ implementation, which required continual bracketing when analyzing the data.

Our multiple case study design was a strength, enabling ICMO comparison across cities, reducing uncertainty, and increasing confidence. Iterative data collection and validation of the program theory with participants further reduced uncertainty because we could discuss uncertainties with participants and dig deeper. We also verified our interpretations with documents.

## Conclusion

We identified broadly applicable insights into the role of TA in strengthening evidence-informed road safety in LMICs and distilled these into a program theory, contributing to knowledge on multisectoral TA programs in global health. Our study is the first we know of to empirically analyze the role of TA in influencing road safety in LMICs. BIGRS’ program theory emphasizes the interaction of trust, credibility, champions and their authority, governance structures, political prioritization, and the implement-ability of evidence in creating the conditions for road safety change. Designing context-specific TA appropriate for structural contexts is critical. If decision makers prioritize road safety, TA can accompany local leaders in adapting international approaches to local realities. In this way, we see cross-country multisectoral projects as important opportunities to improve population health.

### Supplementary Information

**Supplementary Material 1.**

**Supplementary Material 2.**

**Supplementary Material 3.**

## Data Availability

Data generated and analyzed during this study are included in this article. Key informants were assured that the raw transcripts would not be shared.

## References

[1] Institute for Health Metrics and Evaluation. GBD Compare [Internet]. University of Washington. 2019 [cited 2023 Feb 10]. Available from: https://vizhub.healthdata.org/gbd-compare/#

[2] World Health Organisation (2018). Global status report on Road safety 2018 [internet].

[3] Hyder AA, Hoe C, Hijar M, Peden M (2022). The political and social contexts of global road safety: challenges for the next decade. Lancet.

[4] Demystifying the safe system approach [internet]. Vision zero Network. 2023 [cited 2023 Feb 11]. Available from: https://visionzeronetwork.org/resources/demystifying-the-safe-system-approach/

[5] Haghani M, Behnood A, Dixit V, Oviedo-Trespalacios O (2022). Road safety research in the context of low- and middle-income countries: macro-scale literature analyses, trends, knowledge gaps and challenges. Saf Sci.

[6] Shuey R, Mooren L, King M (2020). Road safety lessons to learn from low and middle-income countries. Journal of Road Safety.

[7] Peden MM, Puvanachandra P (2019). Looking back on 10 years of global road safety. Int Health.

[8] Soames Job RF, Wambulwa WM (2020). Features of low-income and middle-income countries making Road safety more challenging. Journal of Road Safety.

[9] West GR, Clapp SP, Averill EMD, Cates W (2012). Defining and assessing evidence for the effectiveness of technical assistance in furthering global health. Glob Public Health.

[10] Dearing JW (2009). Applying diffusion of innovation theory to intervention development. Res Soc Work Pract.

[11] Dearing JW, Cox JG (2018). Diffusion of innovations theory, principles, and practice. Health Aff.

[12] Greenhalgh T, Robert G, Macfarlane F, Bate P, Kyriakidou O (2004). Diffusion of innovations in service organizations: systematic review and recommendations. Milbank Q.

[13] Olivier de Sardan J-P, Diarra A, Moha M (2017). Travelling models and the challenge of pragmatic contexts and practical norms: the case of maternal health. Health Res Policy Syst.

[14] UN Road Safety Fund. Open day knowledge kit. New York; 2023.

[15] International transport forum (2022). The safe system approach in action. Paris.

[16] Turner B, Job S, Mitra S. Guide for Road safety interventions: evidence of what works and what does not work. Washington; 2021.

[17] Sleet D, Baldwin G. Lost in translation: translating injury research into effective interventions. J Australas Coll Road Saf. 2010;

[18] LaJeunesse S, Heiny S, Evenson KR, Fiedler LM, Cooper JF (2018). Diffusing innovative road safety practice: a social network approach to identifying opinion leading U.S. cities. Traffic Inj Prev.

[19] Knapp K, Walker D, Wilson E (2003). Challenges and strategies for local Road safety training and technology transfer. Transportation Research Record: Journal of the Transportation Research Board.

[20] Koon AD, Lopez-Hernandez A, Hoe C, Vecino-Ortiz AI, Cunto FJC, de Castro-Neto MM (2022). Multisectoral action coalitions for road safety in Brazil: an organizational social network analysis in São Paulo and Fortaleza. Traffic Inj Prev.

[21] UN Road Safety Fund [Internet]. United Nations Road Safety Fund. [cited 2024 Mar 1]. Available from: https://roadsafetyfund.un.org/

[22] Global Road Safety Facility [Internet]. World Bank Group. 2023 [cited 2024 Mar 1]. Available from: https://www.roadsafetyfacility.org/

[23] EU international cooperation in road safety [Internet]. European Commission . [cited 2024 Mar 1]. Available from: https://road-safety.transport.ec.europa.eu/what-we-do/eu-international-cooperation-road-safety_en

[24] United Nations Institute for Training and Research. ROAD SAFETY INITIATIVE [Internet]. [cited 2024 Mar 1]. Available from: https://unitar.org/sustainable-development-goals/people/our-portfolio/road-safety-initiative

[25] Initiative for global Road safety [internet]. Bloomberg Philanthropies 2022 [cited 2022 Dec 11]. Available from: https://www.bloomberg.org/public-health/improving-road-safety/initiative-for-global-road-safety/

[26] Wong G, Westhorp G, Manzano A, Greenhalgh J, Jagosh J, Greenhalgh T (2016). RAMESES II reporting standards for realist evaluations. BMC Med.

[27] Pawson R, Tilley N (1997). Realistic evaluation.

[28] Yin RK (1992). The case study method as a tool for doing evaluation. Curr Sociol.

[29] Yin RK (2009). Case study research - design and methods.

[30] Mukumbang FC, Marchal B, Van Belle S, van Wyk B (2018). Unearthing how, why, for whom and under what health system conditions the antiretroviral treatment adherence club intervention in South Africa works: a realist theory refining approach. BMC Health Serv Res.

[31] Greenhalgh T, Pawson R, Wong G, Westhorp G, Greenhalgh J, Manzano A (2017). What realists mean by context; or, why nothing works everywhere or for everyone.

[32] World Bank Country and Lending Groups [Internet]. World Bank. [cited 2021 Jan 31]. Available from: https://datahelpdesk.worldbank.org/knowledgebase/articles/906519-world-bank-country-and-lending-groups

[33] Population density by city, 2014 [Internet]. Our World in Data. [cited 2022 Dec 26]. Available from: https://ourworldindata.org/grapher/population-density-by-city

[34] Motor vehicles per 1000 inhabitants vs GDP per capita, 2014 [Internet]. Our World in Data . [cited 2022 Dec 26]. Available from: https://ourworldindata.org/grapher/road-vehicles-per-1000-inhabitants-vs-gdp-per-capita?tab=table

[35] Greenhalgh T, Pawson R, Wong G, Westhorp G, Greenhalgh J, Manzano A, et al. Retroduction in realist evaluation. Oxford; 2017.

[36] Manzano A (2016). The craft of interviewing in realist evaluation. Evaluation..

[37] Saunders B, Sim J, Kingstone T, Baker S, Waterfield J, Bartlam B (2018). Saturation in qualitative research: exploring its conceptualization and operationalization. Qual Quant.

[38] Gilmore B, McAuliffe E, Power J, Vallières F. Data analysis and synthesis within a realist evaluation: toward more transparent methodological approaches. International journal of qualitative. Methods. 2019;18.

[39] Mirzoev T, Etiaba E, Ebenso B, Uzochukwu B, Ensor T, Onwujekwe O (2020). Tracing theories in realist evaluations of large-scale health programmes in low- and middle-income countries: experience from Nigeria. Health Policy Plan.

[40] Mumbai police traffic control branch, Bloomberg philanthropies initiative for global Road safety. Mumbai Road safety report 2018 key findings [internet]. Mumbai police traffic control branch. Mumbai; 2018 Apr. Available from: https://archive.org/details/mumbairoadsafetyreport2018keyfindings

[41] Mumbai Live Team. Report shows half of road accident casualties in Mumbai in 2018 were pedestrians [Internet]. Mumbai Live. [cited 2023 Feb 15]. Available from: https://www.mumbailive.com/en/transport/mumbai-road-safety-annual-report-2018-shows-half-of-road-accident-casualties-in-mumbai-in-2018-were-pedestrians-40204

[42] Press Trust of India. Mumbai road safety report suggests 22% decline in accident deaths [internet]. India News 2019 [cited 2023 Feb 15]. Available from: https://www.republicworld.com/india-news/general-news/mumbai-road-safety-report-suggests-22-percent-decline-in-accident-deaths.html

[43] Bhatt A, Mascarenhas B, Ashar D. Redesigning One of Mumbai’s Most Dangerous Intersections in 3 Simple Steps [Internet]. TheCityFix. 2019 [cited 2023 Feb 15]. Available from: https://thecityfix.com/blog/redesigning-one-mumbais-dangerous-intersections-3-simple-steps-amit-bhatt-binoy-mascarenhas-dhawal-ashar/

[44] Natu N. LBS Road, 13 other Mumbai junctions set for pedestrian-friendly redesign [internet]. Times of India 2018 [cited 2023 Feb 15]. Available from: https://timesofindia.indiatimes.com/city/mumbai/lbs-road-13-other-mumbai-junctions-set-for-pedestrian-friendly-redesign/articleshow/64673212.cms

[45] Natu N. Mumbai: Times Square experiment at CSMT junction begins [internet]. Times of India. 2019 [cited 2023 Feb 15]. Available from: https://timesofindia.indiatimes.com/city/mumbai/mumbai-times-square-experiment-at-csmt-junction-begins/articleshow/71715425.cms

[46] Minhas G. Mumbai civic body invites urban designers to improve five streets - [Internet]. Governance Now. 2019 [cited 2023 Feb 15]. Available from: https://www.governancenow.com/news/regular-story/mumbai-civic-body-invites-urban-designers-to-improve-five-streets

[47] Singh D. Thirteen-km-stretch of Lal bahadur Shastri Road to be widened, redesigned [internet]. The Indian Express 2018 [cited 2023 Feb 15]. Available from: https://indianexpress.com/article/cities/mumbai/thirteen-km-stretch-of-lal-bahadur-shastri-road-to-be-widened-redesigned-5241735/

[48] AMA-BIGRS to begin road safety enhancement works at Lapaz [internet]. Accra metropolitan Assembly . 2018 [cited 2023 Mar 10]. Available from: https://ama.gov.gh/news-details.php?n=OTkzczkwMnFvMzc4MjI2OTQzNDIxNjJvNW4yM28xbnNxNjE5cDZvbw==

[49] Agbenorsi J, Kwasin J. Police to check speeding on Accra roads [internet]. Graphic Online. 2019 [cited 2023 Feb 16]. Available from: https://www.graphic.com.gh/news/general-news/police-to-check-speeding-on-accra-roads.html

[50] Kanagat N, Chauffour J, Ilunga JF, Yuma Ramazani S, Ovuoraye Ajiwohwodoma JJP, Ibrahim Anas-Kolo S, et al. Country perspectives on improving technical assistance in the health sector. Gates Open Res. 2021;5:141. 10.12688/gatesopenres.13248.1 PMC8847213 35224453

[51] Scott VC, Jillani Z, Malpert A, Kolodny-Goetz J, Wandersman A. A scoping review of the evaluation and effectiveness of technical assistance. Implement Sci Commun. 2022;3:70. 10.1186/s43058-022-00314-1 PMC9238031 35765107

[52] Sahin I (2006). Detailed review of Rogers’ diffusion of innovations theory and educational technology-related studies based on Rogers’ theory. Turk Online J Educ Technol.

[53] Hawkins B, Parkhurst J (2016). The ‘good governance’ of evidence in health policy. Evidence & Policy.

[54] Sewell WH (1992). A theory of structure: duality, agency, and transformation. Am J Sociol.

[55] Long JC, Cunningham FC, Braithwaite J (2013). Bridges, brokers and boundary spanners in collaborative networks: a systematic review. BMC Health Serv Res.

[56] Pelletier D, Gervais S, Hafeez-ur-Rehman H, Sanou D, Tumwine J (2018). Boundary-spanning actors in complex adaptive governance systems: the case of multisectoral nutrition. Int J Health Plann Manag.

