# Supplementary material for: Translating global evidence into local implementation through technical assistance: a realist evaluation of the Bloomberg philanthropies initiative for global Road safety
Source: Global Health. 2024 May 10;20:42. doi: 10.1186/s12992-024-01041-z (PMC11084027; doi:10.1186/s12992-024-01041-z)
Supplement: Supplementary file 2 — Supplementary Material 2. [file 12992_2024_1041_MOESM2_ESM.docx]

## Additional File Two: Realist Evaluation Protocol

**A realist implementation evaluation of global road safety interventions under the Bloomberg Philanthropies Initiative for Global Road Safety**

**Goal and specific aims**

The goal is this study is to systematically identify and analyze implementation lessons from the Bloomberg Philanthropies Initiative for Global Road Safety (BIGRS) Phase II to inform the implementation and scale-up of road safety interventions LMICs.

**Research Questions:**

1. What were the barriers and enablers to the implementation of road safety activities across BIGRS Phase II sites from 2014 – 2019?
   1. How were they similar or different across selected cities?
2. How were these barriers and enablers associated with programmatic outcomes in selected cities?

If successful, this research will improve global understanding on how to facilitate the implementation and scale-up of evidence-based road safety interventions. It will also improve understanding of the ‘critical mix’ of factors that can enable or constrain the implementation of road safety activities and can be used to inform Phase III of BIGRS.

**Expected Outputs:**

1. A practical ‘key takeaways’ resource for policy makers, practitioners, and funders that outlines lessons for the benefit of the broader road safety community. These findings can be disseminated (e.g. via workshops or webinars) with the support of collaborating partner organizations through the BIGRS consortium.
2. A peer-review publication, analyzing the relative enablers and barriers to implementation in BIGRS Phase II cities.

**Background and Significance**

Road traffic crashes account for an estimated 1.35 million deaths a year and are the leading cause of death among those 5 – 29 years of age[1]. The burden of disease from road crashes is unevenly distributed across countries. The incidence of road traffic mortality is three times higher in low-income countries compared to high-income countries (27.5 deaths per 100,000 compared to 8.3 deaths per 100,000) [1]. Taken together, low- and middle-income countries (LMICs) account for 93% of traffic mortalities but only 60% of the world’s registered vehicles[1]. Africa and South-East Asia have the highest rates of death (26.6 per 100,000 and 20.7 per 100,000 respectively); pedestrians, cyclists, and motorbike drivers are the most vulnerable [1].

Since 2004, global awareness and attention to road safety has grown, with increased data on the burden of road traffic crashes and improved understanding of the effectiveness of road safety interventions [2]. However, most existing evidence is from high-income countries, with low-income country studies focused mainly on helmet use. There have been few studies evaluating the effectiveness of interventions in LMICs, despite the importance of contextual factors in the fidelity of interventions[3]. Further, there is a documented implementation gap in road safety interventions in LMIC setting [2].

In response to this global need and the existing evidence gap, Bloomberg Philanthropies founded the Bloomberg Initiative for Global Road Safety (BIGRS). Phase II of BIGRS supported the implementation of proven road safety interventions in 10 cities (Accra, Addis Ababa, Bandung, Bangkok, Bogotá, Fortaleza, Ho Chi Minh City, Mumbai, Sao Paulo, and Shanghai) with a large burden of road traffic injuries and fatalities from 2015 - 2019. BIGRS provided technical support, including embedded advisors in city governments and organizational support/training, directly to city governments. Partner-supported activities to improve road safety (which will be examined in this study) included (1) road safety audits, (2) trainings and capacity building initiatives, (3) support to new policies and enforcement mechanisms, (4) communication and advocacy efforts, and (5) observational studies to measure the prevalence of risk factors. BIGRS investments complemented existing or planned work on road safety at the regional level in some cities where in other cities it spurred new activities. Governments were largely responsible for financing interventions, with BIGRS and partners also contributing to specific activities (e.g., training).

The intermediate outcome of interest was a change in the prevalence of risk factors. A secondary outcome of interest was changes in legislation and/or enforcement mechanisms for road safety. These activities and intermediate outcomes were designed with the goal of reducing injuries and deaths from traffic crashes in the long term.

By the end of Phase II, cities varied considerably in the way they utilized BIGRS support and the implementation of partner-supported activities. Primary observational data further highlighted substantive variation in the uptake and enforcement of risk factors (speed compliance, seatbelts, child restraints, helmets, and drink driving reduction) across study sites.

**Innovation**

BIGRS’ focus to date has been on the road safety interventions themselves, with less focus on evaluating the implementation process. With the commencement of BIGRS Phase III, there is a growing recognition by the project team that systematic learning from past phases is needed.

The project’s experience has also reinforced the notion that there is an implementation gap for road safety interventions and further indicates that implementation-specific evidence from LMICs is required. Lessons from this study inform BIGRS Phase III program design/implementation and the broader field of road safety. Findings may also be informative for other complex, cross-sectoral public health interventions.

**Approach**

This is an action-oriented and pragmatic qualitative review that aims to systematically identify barriers and enablers to the implementation of road safety activities, understand and how these barriers and enablers were associated with varying outcomes observed in participating cities, and outline implications for future implementation and scale-up, both in BIGRS Phase III and more broadly in LMIC contexts. A realist approach with imbedded implementation case studies will be utilized to accomplish the study aims.

The review will focus on the implementation of five main categories of activities that were supported by the BIGRS during Phase II: (1) road safety audits, (2) trainings and capacity building initiatives, (3) support to new policies and enforcement mechanisms, (4) communication and advocacy efforts, and (5) observational studies to measure the prevalence of risk factors.

**Methodological approach**

A realist approach to systematically generate actionable findings for program learning.

Pawson and Tilly’s realist approach will be adapted for this study. This is a pragmatic, theory-driven approach to policy and program review that focuses on developing and testing the context-mechanism-outcome pattern configurations of an intervention[4]. This allows one to systematically (1) develop theories on how an intervention work, (2) form a working hypothesis about the mechanisms necessary to achieve the desired outcomes, (3) interrogate how the program worked in practice and the contextual realities presumed to influence the relative successes and failures of an intervention, and (4) systematically rule out alternative hypotheses[4]. Although it cannot establish causality, the approach can help to, “describe a detailed picture of the causal web that includes the multiple determinants and to categorize these as intervention, underlying mechanism or essential context factor”[5].

The realist approach rests on four assumptions about how programs work. First, programs are theories, whether explicitly illustrated in a theory of change or implicitly developed by those designing the intervention. Second, programs are ideas embedded into broader systems, and they interact with individuals, institutions, interpersonal relationships, and infrastructure within that system. Third, programs are active; they require some engagement with the intervention by the unit of interest. And fourth, programs are open systems that cannot be closed off from broader contextual realities[4]. To understand these dimensions, a context-mechanism-outcome-pattern is identified, which represents an empirical theory for how the program works and what prerequisites are necessary for program success [4].

Realist approaches are well suited to retrospective research that aim to understand implementation complexities from the perspective of those involved in the program. This is compared to implementation fidelity evaluations, in which implementation outcomes are ideally determined prospectively and measured continuously throughout implementation of the intervention[6]. Other studies have indicated that realist approaches are applicable to both programs as well as large complex policy interventions [7].

Case study design as a component of the realist approach

A multi-case study design will be imbedded into the realist approach to deepen understanding of each selected city’s implementation experiences [8]. Case study designs are appropriate when a phenomenon should be researched within its broader context, when there are many variables relevant to the analysis, and when the boundaries between these factors are not clearly defined[9].

Rationale and advantages for imbedding case studies within a realist review

The strength of a realist review is the ability to generate a ‘middle-range theory’ of how a program works in a real-world setting, while a strength of the case study is its depth. The case studies will deepen understanding within the BIGRS consortium on implementation experiences and variability across cities, as well as provide additional thick description to support transferability. Both are needed to satisfy the study objectives. The middle-range program theory aims to improve transferability of findings across cases to inform the implementation and scale-up of road safety interventions in low-and middle-income country cities.

Examples from the literature on embedding case studies within a realist study include a realist evaluation of a mental health and primary care program in England by Byng et al (2005)[10] and an assessment of a complex maternal and child health intervention in Bangladesh by Adams et al (2015)[11]. This study takes a similar approach to the two cited studies, viewing BIGRS Phase II program implementation in each city as a ‘case’, with differing activities and contextual realities impacting the program’s working theory.

**Study Design**

There are three steps in this study aligned with the two research questions (Figure 1). Steps represent sequential components in the data collection and analysis, with some overlap as preliminary data from one step informs the design and data collection in subsequent step. It uses an emergent design, with the data collected and analyzed in one step directly informing the study design of the step following. In addition to the final outputs, each step in the study has a list of associated outputs. This will allow for practical findings to emerge and to be shared within the consortium on an ongoing basis to inform Phase III of the BIGRS.

Figure 1. Overview of the data collection and analysis process


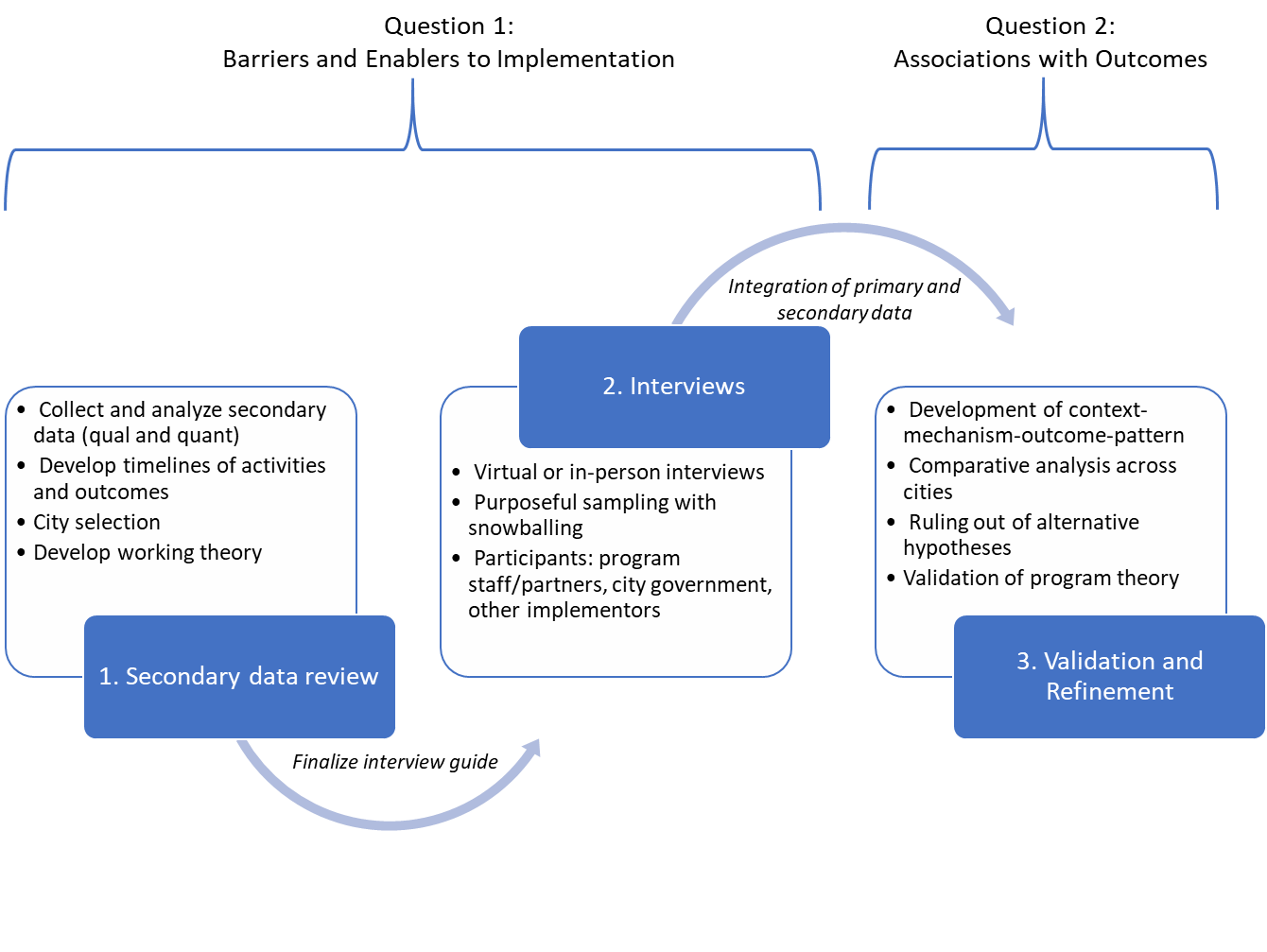


**Step One: Secondary Data Review**

Summary: The goal of step is to increase understanding of BIGRS Phase II, city activities/outputs/outcomes and develop a working theory (or identify the program’s existing theory). The outputs of this step will be: (1) finalizing the definition of success under the program (2) the creation of an initial spectrum of success and ordering of the ten cities along that spectrum; (3) insights needed to develop the interview guide; (4) identification of risk factor(s) for qualitative inquiry; and (5) a basic timeline of each city’s activities, partners, and inputs/outputs/outcomes during Phase II.

Study procedures and conduct

Table 1. outlines a series of working milestones and the study procedures associated with each. This represents a general study plan for step one, with some room for adjustment based on initial findings.

This step will start project-wide and will narrow to selected city study cities by the end of step one. 3-4 cities will be selected for in-depth cases. Geographic diversity and city size will ideally be balanced across the selected. This provides a broader and potentially more transferable understanding of many implementation components.

Table 1. Milestones and components of step one for the realist evaluation

| Milestone | Components |
| --- | --- |
| Develop working program theory (or examine and adapt existing TOC or develop one from existing materials) | - Begin or refine existing TOC/hypothesis via program M&E documentation - Code secondary data, further refine mechanisms, context, and outcome configurations - Develop a series of rapid ‘city profiles’ with salient features of the city (or adapt existing ones as available), its institutions, political environment, capacities, and other ‘outer setting’ details that may have facilitated or hindered implementation |
| Develop spectrum of success and timeline of Phase II | - Utilizing secondary data and consultation with BIGRS consortium staff, develop a spectrum of success for the project vis a vis working theory via collaborative workshop (as possible) - Develop timeline of project activities and layer city activities and outcomes along the timeline - Initial ordering of cities along a spectrum of success |
| City selection | - Finalize boundaries of city selection - Selection of cities for further inquiry |
| Secondary data review for cities | - Conduct second round of data collection and analysis specific to selected cities - Refine city specific timelines and outcomes - Preliminary identification of possible barriers and enablers |
| Refine working theory | - Revisit working theory for relevance across cities, refine as needed |
| Validate working theory | - Validation session with project stakeholders to validate theory and make any final adjustments |

Data Sources

Secondary data will be identified per the considerations in Table 2. This represents an ‘ideal’ starting point, with the understanding that not all documents will be available for all cities.

Table 2. Secondary data collection protocol

| Data Source | Information to Identify |
| --- | --- |
| Project Reports | How the project progressed, activities, successes/challenges, and milestones |
| Meeting Notes | Internal discussions, decisions, and reflections on the project from team members |
| Workshop/training Materials | Activities the project undertook and participants |
| Project Proposal | Intended activities, outputs, and outcomes from the onset of the project |
| Internal presentations | Internal project discussions, debates, and materials on how activities were progressing |
| Field notes and reports | Real-time reflections from project staff on the project’s implementation |
| Program publications | Outcomes from the project |
| Observational data on risk factors and related analysis | Outcomes associated with project activities |
| M&E data | Outcomes associated with project activities |
| Partners/sub-awardee reports and budgets | Activities of partner organizations, successes/challenges, and milestones |
| Outcome data for fatalities and injuries | Outcomes associated with project activities |
| Communication materials | From the project and outside the project (e.g. international or local media) to see how project activities were represented and outcomes communicated |
| Social network analysis (SNA) | Interview data from the SNA will be re-coded under the framework for this review |

Data Analysis

For the qualitative document review, a preliminary review of the documents will be completed and a refined list of relevant documents for the research question will be catalogued accordingly. Documents will be coded inductively to facilitate the development of the initial mechanisms, context, and outcome configurations. Atlas.ti or a similar qualitative data software will be utilized to facilitate this process. The use of a research memo will facilitate reflexivity and the identification of further questions for the project team.

Quantitative data has already been cleaned and analyzed during the Phase II implementation. Emphasis will therefore be on reviewing the findings of the outcome data (fatalities and injuries) and the observational risk factors (seat belt use, helmets, child restraint, speed, and drink driving).

Sample size

Initially, all project-wide documents will be collected. Once city selection is finalized are selected, a second round of data collection and review will take place specific to the selected city sites. The expected sample of cities is 3 – 5, dependent on the findings from step one regarding the ordering of cities and feasibility/timelines for data collection and analysis in subsequent phases.

**2. Step Two: Testing of program hypotheses**

Summary: The goal of this step is to deepen understanding of barriers and enablers to implementation in 3-4 cities (including analysis of similarities and differences). The output of this step will be a series of mini-implementation case studies, one per city.

Study procedures and conduct

Table 3. outlines a series of working milestones and the study procedures associated with each. This represents a general study plan for step two, with some room for adjustment based on initial findings. During this phase, all interviews, inclusive of respondents and questions asked, will be coordinated with a complementary social network analysis being conducted by BIGRS to prevent duplication and reduce burden on respondents.

Interviews within the BIGRS consortium and its collaborators will be conducted virtually over Zoom. Ideally, city interviews will be done in-person by a team of two people (from JHU and a local partner/collaborator). If travel is not feasible due to COVID-19 safety and travel restrictions, city interviews will be conducted through a combination of Zoom and in-person interviewing by BIGRS staff/partners based in the city. Virtual training would be conducted prior to data collection via Zoom.

Table 3. Milestones and components of step two

| Milestone | Components |
| --- | --- |
| Develop interview guide(s) | - Drawing on working hypothesis from step one, refine and finalize the initial interview guide. - Questions inclusive of program experiences and how the broader context (e.g., political environment, capacities, institutional structure) interacted with aspects of implementation |
| Develop initial list of study participants | - Identify profiles of informants - Discussions with BIGRS consortium, inclusive of city managers, to finalize individual participants |
| Test interview guides | - Review of guides with program staff that are knowledgeable about interview participants. - Testing of guide with sample interview participant - Adjustments to guide and finalization |
| Conduct interviews | - Allow time for some snowballing of data collection as relevant |
| Transcription | - 1-2 weeks for transcription service |
| Data analysis |  |

Data Analysis

Interviews will be coded utilizing Atlas.ti or similar qualitative data software. A blended deductive/inductive approach will be taken to facilitate coding. Preliminary codes will be developed utilizing the working program theory, developed inductively from secondary data in step one. As transcripts are coded, additional codes will be identified inductively from the data and added to the codebook as required.

Utilizing the coded primary data, barriers and enablers will be identified for each city. These will then be compared across sites to identify commonalities and differences. Findings will be triangulated both across participants working in the same city, and across the primary and secondary data. Divergent findings will be discussed with the project team and if needed, brief follow-up inquiry will be pursued to fully understand divergent views.

Sample size

Purposeful sampling will be utilized to select study participants. Approximately 35 - 50 interviews, across 3-5 cities will be conducted, depending on the number of cities selected for case studies, the extent of secondary data identified, and saturation. Depending on scheduling and the preferences of participants, group interviews may be utilized in addition to individual interviews. A sample interview list and inclusion/exclusion criteria, to be further developed and finalized during the study, is found in Table 4. Flexibility will be allowed for limited snowball sampling if participants identify other key implementation actors that were not considered in the initial list of participants.

Table 4. Proposed study participants

| Participant type | Inclusion Criteria | Exclusion criteria | Estimated # |
| --- | --- | --- | --- |
| BIGRS JHU IIRU Staff | - Employed during BIGRS Phase II, ideally during the program’s duration - Day-to-day involvement with one or more program activities | - Hired after Phase II - Operational role that did not engage with activity implementation | 3 |
| BIGRS Global Partners and Collaborators | - Either: (i) worked directly with BIGRS Phase II staff or activities or (ii) worked with the same government counterparts as BIGRS on a road safety program or related initiative | - Road safety professional that was not engaged, directly or indirectly, in BIGRS’ work | 5 |
| BIGRS in-country partners in selected cities | - Employed by a contracted or collaborating organization based locally/regionally in a BIGRS city during BIGRS Phase II - Day-to-day involvement with one or more program activities | - Hired after Phase II - Operational role that did not engage with activity implementation | ~ 5 per city |
| City managers | - Day to day counterpart person for BIGRS Phase II in the selected city |  | 1 -2 per city |
| Government officials | - Government official whose work includes road safety, broadly defined, in the city of interest - (i) employed in the legislative or executive branches of city government or (ii) employed at the state/district or national level, if they were engaged in work relevant to city-level implementation - Active role in road safety during the BIGRS Phase II - Ideally, participated in one or more BIGRS Phase II activities | - National officials whose work did not have the potential to impact the city of interest - Officials whose mandate includes road safety but have not engaged with any road safety activities or policies - Persons who do not work in government | 2 - 3 per city |
| City-level implementers | - Participated actively in BIGRS Phase II activities; ideally, led or helped to organize activities - National counterparts at the implementation level for BIGRS Phase II activities - Direct mandate for aspects of road safety in the city during Phase II | - Counterparts employed by BIGRS or partners (see BIGRS staff) - Not employed or not working in road safety during the Phase II period | 3-5 per city |

Step Three: Refinement of program theory and testing of alternative hypotheses

Summary: The goal of this step is to interrogate the extent to which the implementation barriers and enablers identified in the city studies fit cohesively with the working program theory and to test alternative explanations for the outcome(s) of interest. Details on each city’s contextual reality (the ‘outer setting’) from phase one will also be examined vis a vis the phase two interview findings to deepen understanding of how the program theory interacts with the broader system at the city level. The outputs of this step will be: (1) an additional layer of comparative case analysis, (2) the finalized program theory and cross-city learnings, and (3) implications/recommendations for future implementation and scale-up of road safety interventions.

Study procedures and conduct

Table 5. outlines a series of working milestones and the study procedures associated with each. This represents a general study plan for step three, with some room for adjustment based on initial findings. This stage is expected to be particularly iterative, as findings from the comparative city analysis inform the program theory and adjustments to the program theory require another round of primary data analysis. This ‘cycle’ is expected to require several iterations until a finalized program theory is validated with stakeholders.

Table 5. Milestones and components of step three

| Milestone | Components |
| --- | --- |
| Comparative analysis of cities | - Commonalities and differences examined across each city |
| Refinement of working theory | - Comparative analysis of each city experience against working theory - Refine/adjust working theory as needed to encompass city experiences |
| Alternative hypothesis testing |  |
| Development of recommendations | - Drawn from program theory and other findings from primary and secondary data analysis |
| Validation with program stakeholders | - Discussion and validation - Final adjustments to program theory |
| Finalization |  |

Strengths and Limitations

This is a retrospective study, and the presence of data is a limitation. It is possible that data will be differentially available based on many factors, including the outcome variable of interest (implementation ‘success’). Cities that were not particularly engaged in the BIGRS Phase II program may have less data available for review and are therefore less likely to be selected for the city studies.

An additional limitation of this study is retrospective interviewing. Participants may have recall errors, especially for activities that took place in the earlier years of the program (e.g., 2014 – 2016). Bias may be introduced in the ability to contact past stakeholders, including those who have left the BIGRS study team, collaborating partner organizations, and/or city governments. It is possible that stakeholders who are not available for interview look systematically different than those who have remained working with, or who can be reached by, BIGRS project staff.

Conversely, conducting a retrospective study also has its strengths. Should data availability be sufficient, the study will be able to trace implementation pathways from beginning to end because the final data on city outcomes is available. While this can be done in a prospective study, it would take several years to complete. Doing this retroactively for BIGRS Phase II allows for more rapid data collection and analysis, which can then directly inform BIGRS Phase III implementation.

The nesting of this study within BIGRS Phase III is both a limitation and a strength. The study will have access to all prior secondary data from Phase II, inclusive of meeting notes, reports, and outcome data. This makes the retrospective nature of the research more feasible and should mitigate some of the limitations described above. However, project stakeholders who are engaged in BIGRS Phase III may be more likely to provide positive responses to implementation questions to confirm or add to perceptions that their city/organization has performed well throughout the project. To limit this, the interviewers will need to maintain neutrality as much as possible from the current implementation and especially any decisions regarding project milestones and timelines. Confidentiality of transcripts will need to be maintained, even from general members of the BIGRS project team. Data from the individual interviews will be triangulated from secondary data, especially project notes and reports, to ensure a level of rigor and accuracy in the analysis of interview data.

**References**

1. World Health Organisation. Global Status Report on Road Safety 2018 [Internet]. World Health Organization. Geneva; 2018. Available from: http://apps.who.int/bookorders.

2. Hyder AA, Allen KA, Di Pietro G, Adriazola CA, Sobel R, Larson K, et al. Addressing the Implementation Gap in Global Road Safety: Exploring Features of an Effective Response and Introducing a 10-Country Program. Am J Public Health. 2012;102:1061–7.

3. Hyder AA, Allen KA, Peters DH, Chandran A, Bishai D. Large-scale road safety programmes in low- and middle-income countries: An opportunity to generate evidence. Glob Public Health [Internet]. 2013 [cited 2020 Apr 13];8:504–18. Available from: http://www.tandfonline.com/doi/abs/10.1080/17441692.2013.769613

4. Pawson R, Tilley N. Realistic evaluation. Newbury Park: Sage Publications, Inc.; 1997.

5. Marchal B, Dedzo M, Kegels G. A realist evaluation of the management of a well- performing regional hospital in Ghana. BMC Health Serv Res. 2010;10:24.

6. Proctor E, Silmere H, Raghavan R, Hovmand P, Aarons G, Bunger A, et al. Outcomes for implementation research: Conceptual distinctions, measurement challenges, and research agenda. Administration and Policy in Mental Health and Mental Health Services Research [Internet]. 2011 [cited 2021 May 2];38:65–76. Available from: https://www.ncbi.nlm.nih.gov/pmc/articles/pmid/20957426/?tool=EBI

7. Pedersen LH, Rieper O. Is Realist Evaluation a Realistic Approach for Complex Reforms? Evaluation [Internet]. 2008 [cited 2020 May 2];14:271–93. Available from: http://journals.sagepub.com/doi/10.1177/1356389008090856

8. Yin RK. The Case Study as a Serious Research Strategy. Knowledge [Internet]. 1981 [cited 2022 Oct 5];3:97–114. Available from: https://journals.sagepub.com/doi/epdf/10.1177/107554708100300106

9. Yin RK. Case Study Research - Design and Methods. 4th ed. Thousand Oaks: SAGE Publications; 2009.

10. Byng R, Norman I, Redfern S. Using Realistic Evaluation to Evaluate a Practice-level Intervention to Improve Primary Healthcare for Patients with Long-term Mental Illness. Evaluation. 2005;11:69–93.

11. Adams A, Sedalia S, McNab S, Sarker M. Lessons learned in using realist evaluation to assess Maternal and Newborn health programming in rural Bangladesh. Health Policy Plan. 2016;31:267–75.
