# Supplementary material for: Translating global evidence into local implementation through technical assistance: a realist evaluation of the Bloomberg philanthropies initiative for global Road safety
Source: Global Health. 2024 May 10;20:42. doi: 10.1186/s12992-024-01041-z (PMC11084027; doi:10.1186/s12992-024-01041-z)
Supplement: Supplementary file 3 — Supplementary Material 3. [file 12992_2024_1041_MOESM3_ESM.docx]

## Additional File Three: Expanded documentation of revised program theory development

This supplement provides a detailed summary of the process of constructing the Bloomberg Philanthropies Initiative for Global Road Safety (BIGRS) program theory.

### Boundaries of the study

The boundaries of this study were the intermediate outcomes that were achieved or intended to be achieved via direct recipients of the intervention (including city government agency staff, police, and city engineers). BIGRS interventions that addressed broader behavioral change of drivers (e.g., mass media and communications campaigns) or vehicle safety were considered out of scope.

### Describing the initial program theory – the BIGRS Theory of Change

As is common in this literature[1], BIGRS’ theory of change (TOC) was used as the initial program theory (IPT) (Figure 1).

The TOC was developed retrospectively at the end of BIGRS Phase II. It outlines the hypothesized linkages between BIGRS’ interventions, the five risk factors that BIGRS was focused on, and BIGRS’ goal of reducing traffic-related injuries and deaths. The authors of this study, in collaboration with other BIGRS staff, were involved in the development of the TOC. The TOC was finalized prior to the start of the realist evaluation and was not a component of the realist evaluation.


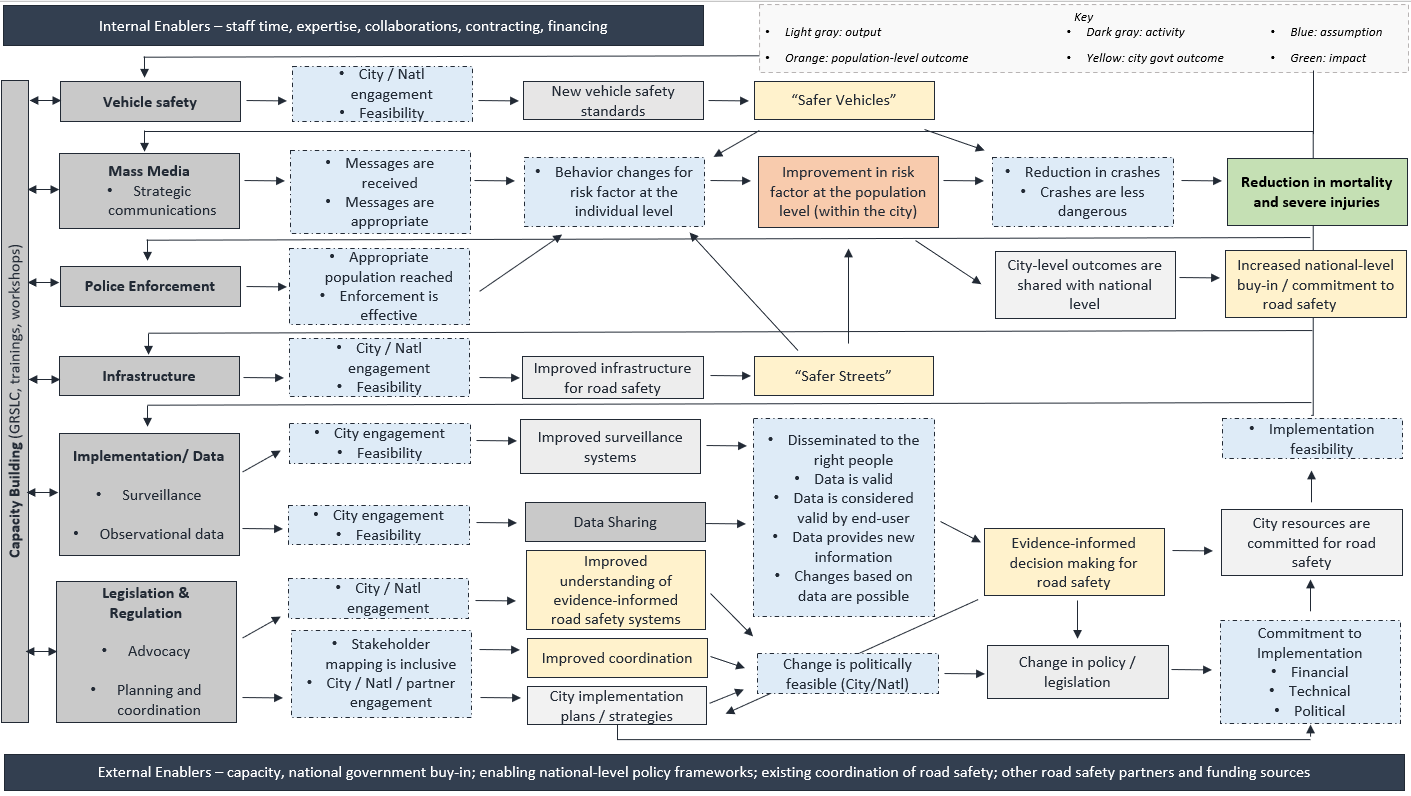
Figure 1. BIGRS Theory of Change

### Connecting the Initial Program Theory (IPT) and diffusion of innovation theory

Building a program theory requires iteratively refining the IPT alongside both empirical data and theory. To select candidate theories from the literature, we followed advice from Shearn et al. on theory selection by asking, ‘what is BIGRS principally an example of?’ This was used to guide candidate theory selection[2]. We considered that BIGRS was ‘a planned effort to translate a technical concept from elsewhere to somewhere new’ – which led us to initially apply diffusion of innovation theory and policy diffusion and transfer theories. These theories provided working hypotheses about casual processes and contextual conditions and were used to iteratively refine the program theory presented in the results section [3]. Theories were iteratively reviewed, selected, and discarded throughout the analysis process, as we continually learned more about the program and how it worked vis a vis primary and secondary data collection [1,2]. Ultimately, diffusion of innovation theory [4]and the related Greenhalgh’s model for the diffusion of innovations [5]were the most applicable theories to explain the results across all three cases.

### Data Collection

Initial theory gleaning interviews were conducted with BIGRS staff at the city level [6]. These interviews tested our understanding of how the program worked and were used to refine how the context, interventions, potential mechanism, and outcomes were the same or different across the three cases.

Next, we conducted theory refinement interviews[6] with city government informants, partner organizations, and global staff that were engaged in BIGRS. We iteratively triangulated information across these sources as we gathered new information [6]. For example, if partners in a specific city spoke about improved focus on pedestrian safety, we probed government informants on how they defined road safety to see if they emphasized pedestrians. We also consulted government documents and news articles to see if pedestrians were mentioned. As insights on possible ICMOs developed, we began testing these configurations with participants by asking them to agree, disagree, or refine my interpretations of how program outcomes were achieved. We then compared findings across cities to deepen our understanding of how context was differentially impacting the outcomes.

Finally, we conducted theory consolidation interviews [6]with global staff familiar with the case study cities as well as other contexts where BIGRS worked. This allowed us to refine the working theory further by understanding how the context of the case study cities influenced construction of the ICMO and sought to confirm or disprove the working ICMOs. Table 1 explains how a realist approach to interviewing was used for the data collection and provides example interview questions.

Table 1. Realist approach to interviewing and example informants and interview questions

| **Phase of realist interviewing** | **Purpose** | **Type of informant interviewed** | **Example interview questions^1^** |
| --- | --- | --- | --- |
| Theory gleaning | Build our understanding of how then program worked | Embedded staff and staff of international partner organizations working specifically in the three case study cities | - What were the major accomplishments of BIGRS 2014 to 2019? - Probe for barriers, enablers, specific actors and their roles, how things changed over time. Ask about underlying mechanisms – why specifically did that occur? What |
| Theory refinement | Test various ‘hunches’ on how the interventions, context, mechanisms, and outcomes interacted | City government informants, partner organizations, and global staff | - I know BIGRS supported infrastructure redesign projects in Mumbai. How did that process relate to existing government process for selecting junctions for improvement and/or maintenance? - I’ve heard from others that BIGRS did a lot of trainings that improved understanding of the safe systems approach. What specific things about those trainings led to an improved understanding? Can you give an example? |
| Theory consolidation | Understanding how the context of the case study cities influenced our construction of the ICMO and to confirm, refine, or refute our working ICMOs | International organization staff familiar with both the case study cities and other BIGRS cities | - In the three cities of focus for this study, government staff turnover has repeatedly come up as a major challenge. Is this something you have experienced in the cities where you work? How has it influenced BIGRS’ activities? Can you give an example? |
| Theory validation  (not completed) | Refined program theory is presented to participants and further refined | Planned as a continuation of this study |  |

### Developing the ICMOs

The context-mechanism-outcome (CMO) heuristic forms the analytical basis for realist evaluation. As described in the main text, we adopted the intervention component, expanding from CMO to an ICMO.

The process of constructing the ICMO was iterative and moved across both data sources. An initial codebook was developed using components of the IPT. This was modified after coding an initial set of transcripts because study participants were describing interconnected, intermediate outcomes that required extensive double coding against the IPT. For this reason, we did an initial round of inductive coding focused on identifying outcome themes that were described by participants [1], while continuously triangulating the outcomes identified with the IPT.

To maintain fidelity to how the participants described possible ICMOs, we first coded large segments of text under the described outcome, avoiding the splitting of data into ICMO components at the early stage. Cross-cutting contextual factors that were linked to multiple outcomes were coded as overall barriers and enablers. Coding was initially completed for each case separately, with the goal of deriving separate program theories for each case and then conducting a cross-case synthesis [7]. However, after coding over 50% of the KII data from each city, the themes in each city were similar, so we merged the three datasets (Accra, Bogotá, Mumbai). This process was the theory gleaning phase.

For theory refinement, we divided coded data segments further into working contexts, mechanisms, and outcomes and documented the process in memos [8]. Retroductive analysis was used to continuously identify and challenge patterns in the data and to look for underlying forces at work [7,9]. As potentially causal patterns were identified, we sought to further refine or dispute them through triangulation with data from other BIGRS-supported cities. We also iteratively compared the emerging ICMOs to the initial set of social science theories chosen for the evaluation as a means by which to challenge our data and assumptions and to guide us on how we might test our emerging hypotheses.

### Abstracting six ICMOs into the two program theories

Initially, six ICMOs were developed for each of BIGRS program outcomes as stated in the TOC – (1) improved understanding of road safety through a safe systems approach, (2) increased coordination of road safety agencies, (3) increased use of data to target road safety interventions, (4) safer streets via improvements to the built environment/ physical road infrastructure, and (5) strengthened evidence-based enforcement approaches – and cross-cutting barriers and enablers identified across the ICMOs. These are described in more detail in Table 2.

Table 2. Working version of the expanded ICMO configurations (drawn from memos during the analysis process)

| **Outcome** | **Intervention** | **Context** | **Mechanisms** | **Relationship to the other ICMOs** |
| --- | --- | --- | --- | --- |
| **Improved understanding of road safety through a safe systems approach** | - Trainings - Direct technical inputs | - If supported by high level leadership, and in a context of an open mind - If the training is practical, relevant, locally-adapted, and facilitative of local perspectives, capabilities, and skills | - Shifts perspective - Builds confidence | - Supported by training and capacity building evaluations - Supports all others, but especially (only) if senior leaders are engaged |
| **Data use** | - Surveillance data management and analysis - Observational studies / prevalence of risk factors - Infrastructure assessments | - In an environment where this information is considered both novel and if the dissemination includes the appropriate set of stakeholders who are able and willing to act on the findings - And if introduced within a broader environment of TA support – unclear | - Changes perception about the problem | - Supports all other outcomes - Improved coordination supports data use because data is from police but used by other agencies |
| **Enforcement** | - Pilot - Equipment | - If the police have the necessary leadership support/directives, equipment, and training, are supported with a legal and administrative framework to make decisions, if the existing social system allows for enforcement, and if the TA provider has built enough trust with the police to allow them to provide the intervention | - Builds confidence - Risk – connection to societal perception | - Requires improved understanding. - Supported by use of data |
| **Infrastructure** | - Pilot - Input into existing city projects | - If the city has authority over the built environment to authorize intervention, if the approaches are considered low-cost and feasible within existing resources, and if the pilot is considered a success via data collected by the initiative. | - Builds confidence - Reduces risk | - Supported by improved understanding, use of data, coordination |
| **Policy change** | - Pilot - Data use - Coordination - Leadership | - If city leadership prioritizes road safety, has the authority to act, can effectively coordinate across stakeholders. | - Shifts perspective - Builds confidence - Reduces risk | - Conglomeration of other ICMOs; required multiple to come together. |

Translating the six ICMOS into the two program theories was done via a process of abstraction that identified demi-regularities – a semi-predictable pattern – across the cases and the ICMOs.

From the TOC to the program theory – how the theory evolved

The program theory is designed to a middle-range theory – close enough to the data but abstracted enough to be testable [10]. Several rounds of abstraction to look for ‘demi-regularities’[11] across different ICMOs transformed the IPT’s six intervention categories and outcomes into two intervention categories and outcomes (strengthening road safety capabilities and increasing evidence-informed implementation).

The biggest change from the TOC to the program theory is the centering of individuals and their agency. BIGRS itself is not reflected in the TOC, but the program theory places BIGRS directly into the context by considering how the interventions were delivered and who delivered them.

Relatedly, while city engagement is an assumption on the TOC, the program theory provides a richer description of the city officials’ agency and their importance to bringing about the interventions. For example, the TOC implies that: ‘*infrastructure activities, if the city or national government is engaged and if the change is feasible, will improve infrastructure’*. Based on the program theory, it would be more appropriate to expect that ‘*if the city government is engaged and pushes forward the work alongside BIGRS TA providers, then infrastructure activities can be implemented, building confidence, and lowering risk, thereby improving infrastructure but only within the scale of individual’s authority and potentially only during city official’s tenure’*.

Strengths, limitations, uncertainties, and interpretation of the findings and application of the program theory

The program theory developed from this study aims to do two things. First, it prioritizes the interventions and the contexts that were consistently important to understanding the outcomes (or lack thereof) across the three cities. This can guide efforts, from BIGRS or other funding partners, to design more context-specific TA programs in the future. Second, it provides a working set of plausible explanations of how BIGRS can strengthen evidence-informed road safety. This can be validated, refined with a wider set of stakeholders, and empirically tested in BIGRS Phase III.

Sources of uncertainty emerged in the data collection and analysis that are ripe for future refinement. For example, limited programmatic monitoring and evaluation (M&E) documents was a limitation, as it prevented us from further characterizing or evaluating the outcomes. This introduces some uncertainty; for example: were outcomes different because cities prioritized different interventions? Or were outcomes different because the context differentially impacted the hypothesized mechanisms? Because of our iterative data collection and analysis, we brought these types of questions back to our informants and discussed them in detail as we constructed the ICMOs.

Future work should explicitly consider potential alternative mechanisms to explain the outcomes. Testing the theory alongside implementation in Phase III could improve the inclusion of city government officials and further refine the program theory.

**References**

1. Mirzoev T, Etiaba E, Ebenso B, Uzochukwu B, Ensor T, Onwujekwe O, et al. Tracing theories in realist evaluations of large-scale health programmes in low- and middle-income countries: experience from Nigeria. Health Policy Plan. 2020;35:1244–53.

2. Shearn K, Allmark P, Piercy H, Hirst J. Building Realist Program Theory for Large Complex and Messy Interventions. Int J Qual Methods. 2017;16:160940691774179.

3. Van Belle S, Van De Pas R, Marchal B. Towards an agenda for implementation science in global health: There is nothing more practical than good (social science) theories. BMJ Glob Health. 2017;2:e000181. Available from: http://dx.doi.org/10.1136/

4. Sahin I. DETAILED REVIEW OF ROGERS’ DIFFUSION OF INNOVATIONS THEORY  AND EDUCATIONAL TECHNOLOGY-RELATED STUDIES BASED ON ROGERS’ THEORY. The Turkish Online Journal of Educational Technology. 2006;5:1303–6521.

5. Greenhalgh T, Robert G, Macfarlane F, Bate P, Kyriakidou O. Diffusion of Innovations in Service Organizations: Systematic Review and Recommendations. Milbank Q. 2004;82:581–629.

6. Manzano A. The craft of interviewing in realist evaluation. Evaluation. 2016;22:342–60.

7. Gilmore B, McAuliffe E, Power J, Vallières F. Data Analysis and Synthesis Within a Realist Evaluation: Toward More Transparent Methodological Approaches. International Journal of Qualitative Methods. 2019;18. Available from: https://journals.sagepub.com/doi/full/10.1177/1609406919859754

8. Dalkin S, Forster N, Hodgson P, Lhussier M, Carr SM. Using computer assisted qualitative data analysis software (CAQDAS; NVivo) to assist in the complex process of realist theory generation, refinement and testing. Int J Soc Res Methodol. 2021;24:123–34.

9. Greenhalgh T, Pawson R, Wong G, Westhorp G, Greenhalgh J, Manzano A, et al. What realists  mean by context; or, why nothing works everywhere or for everyone. . 2017.

10. Merton R. Social theory and social structure. New York: Free Press; 1968.

11. Mukumbang FC, Marchal B, Van Belle S, van Wyk B. Unearthing how, why, for whom and under what health system conditions the antiretroviral treatment adherence club intervention in South Africa works: A realist theory refining approach. BMC Health Serv Res. 2018;18:343.
